# Supplementary material for: Anti-Obesity Effects and Changes of Fecal Microbiome by Lactic Acid Bacteria from Grains in a High-Fat Diet Mouse Model
Source: Int J Mol Sci. 2025 Sep 17;26(18):9056. doi: 10.3390/ijms26189056 (PMC12471125; doi:10.3390/ijms26189056)
Supplement: Supplementary file 1 [file ijms-26-09056-s001.zip › ijms-3832805-supplementary.pdf]

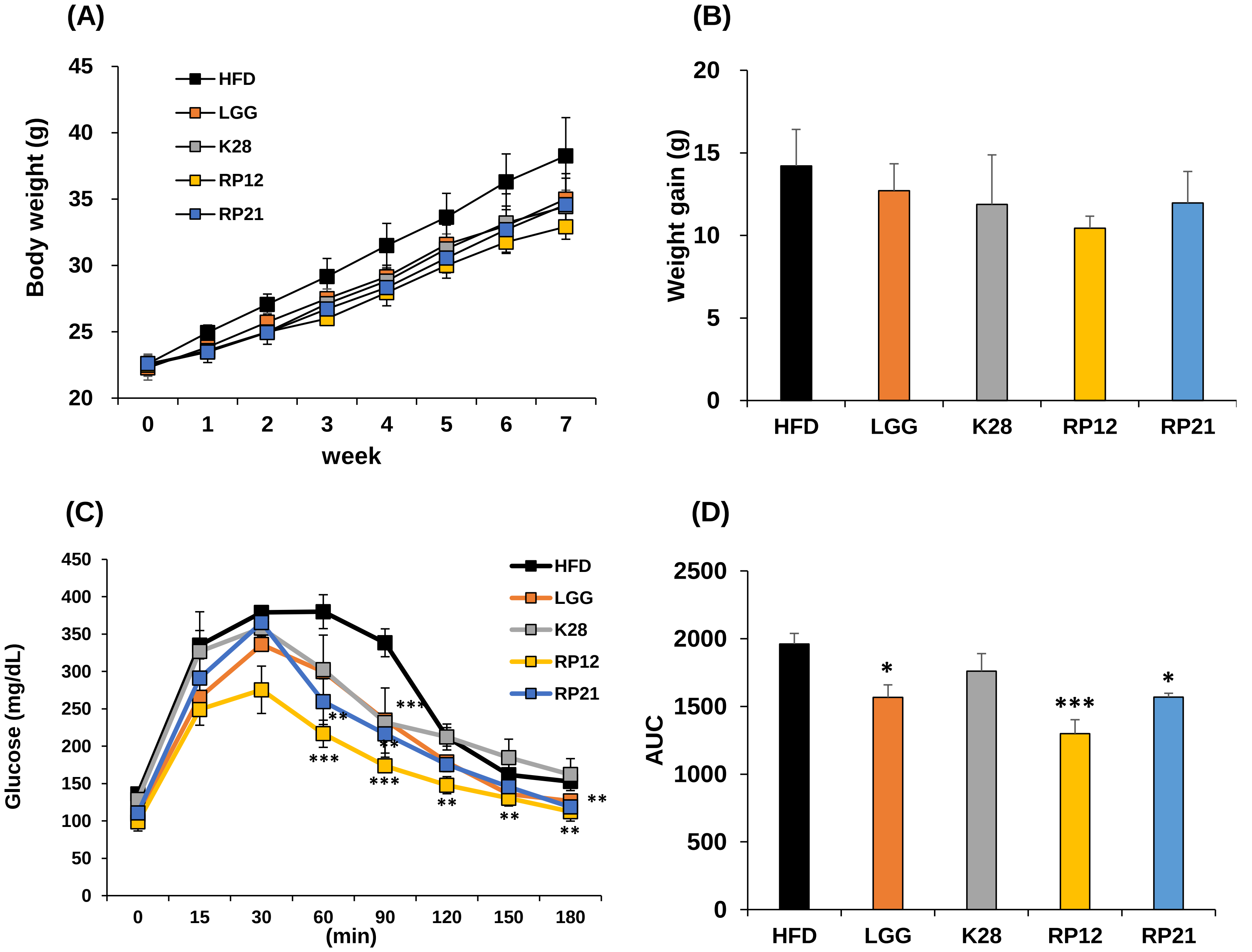

Supplementary Figure 1. Effects of *Lacticaseibacillus rhamnosus* GG, *Pediococcus pentosaceus* K28, *Lactiplantibacillus plantarum* RP12 and *Levilactobacillus brevis* RP21 on high fat diet-induced obese mouse model. (A) Weight change of 5 groups over 7 weeks. (B) Total weight gain for each group after 7 weeks. (C) Glucose tolerance test. (D) Area under curve. Mice were fasted for 12 h before intraperitoneal injection of glucose (2 g/kg). Results are shown as mean ± SEM (n = 3). Significant difference between groups are indicated as *$p < 0.05$, **$p < 0.01$, *** $p < 0.001$

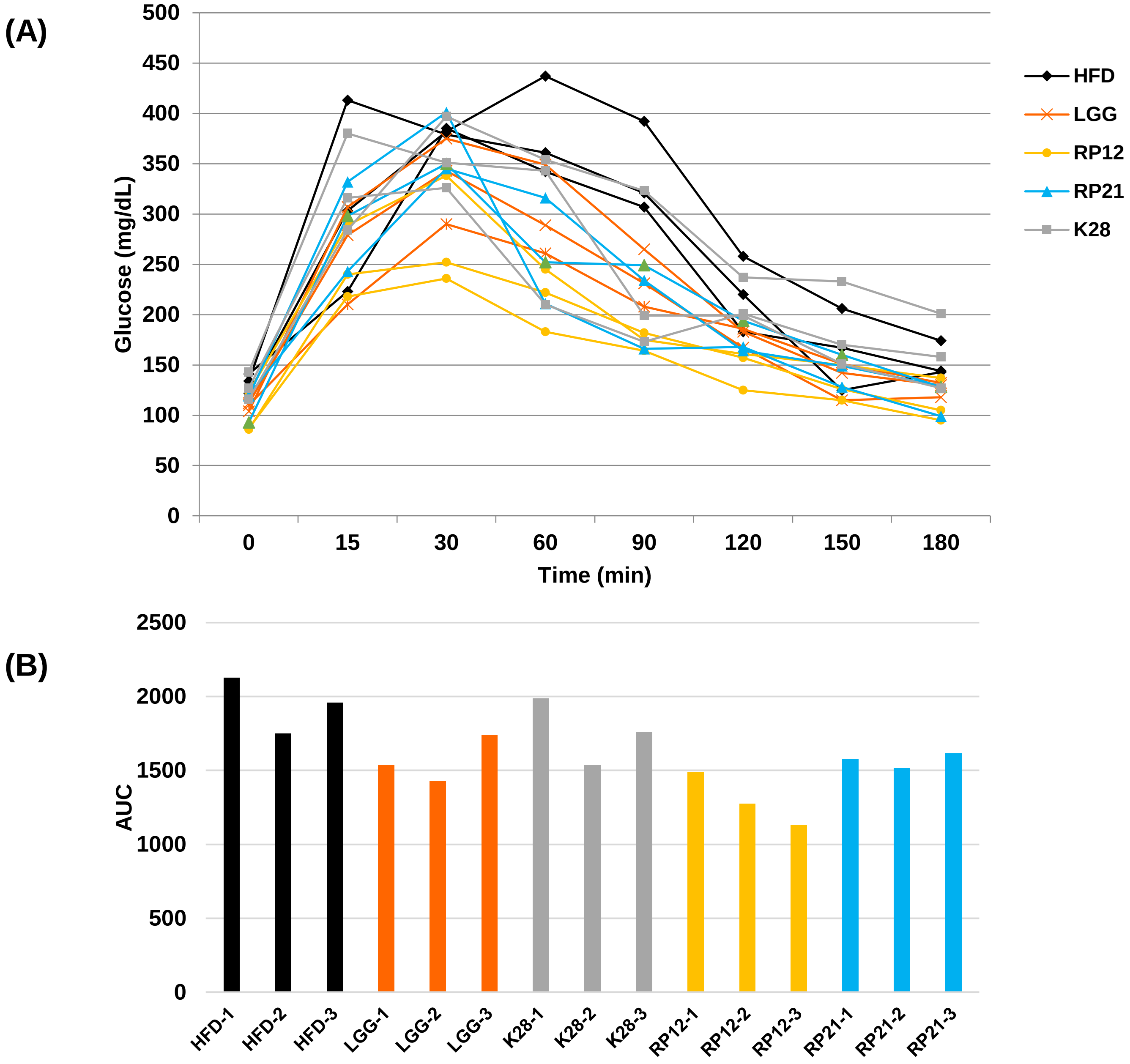

Supplementary Figure 2. Effects of *Lacticaseibacillus rhamnosus* GG, *Pediococcus pentosaceus* K28, *Lactiplantibacillus plantarum* RP12 and *Levilactobacillus brevis* RP21 on high fat diet-induced obese mouse model. (A) Glucose tolerance test for individual mouse. (B) Area under curve for individual mouse. Mice were fasted for 12 h before intraperitoneal injection of glucose (2 g/kg).

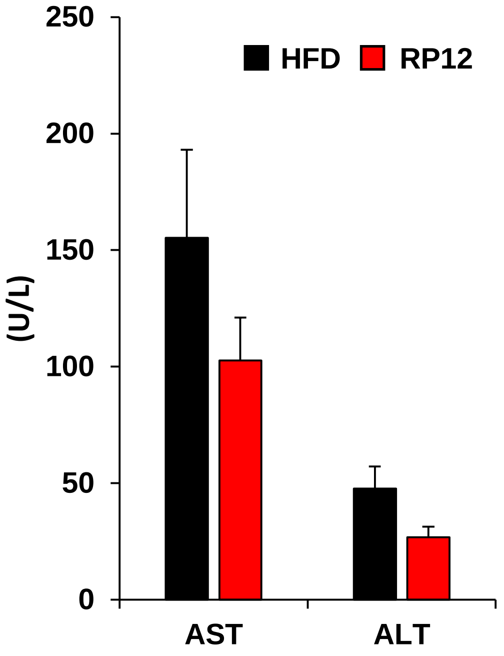

Supplementary Figure 3. Effects of *Lactiplantibacillus plantarum* RP12 on serum AST and serum ALT.

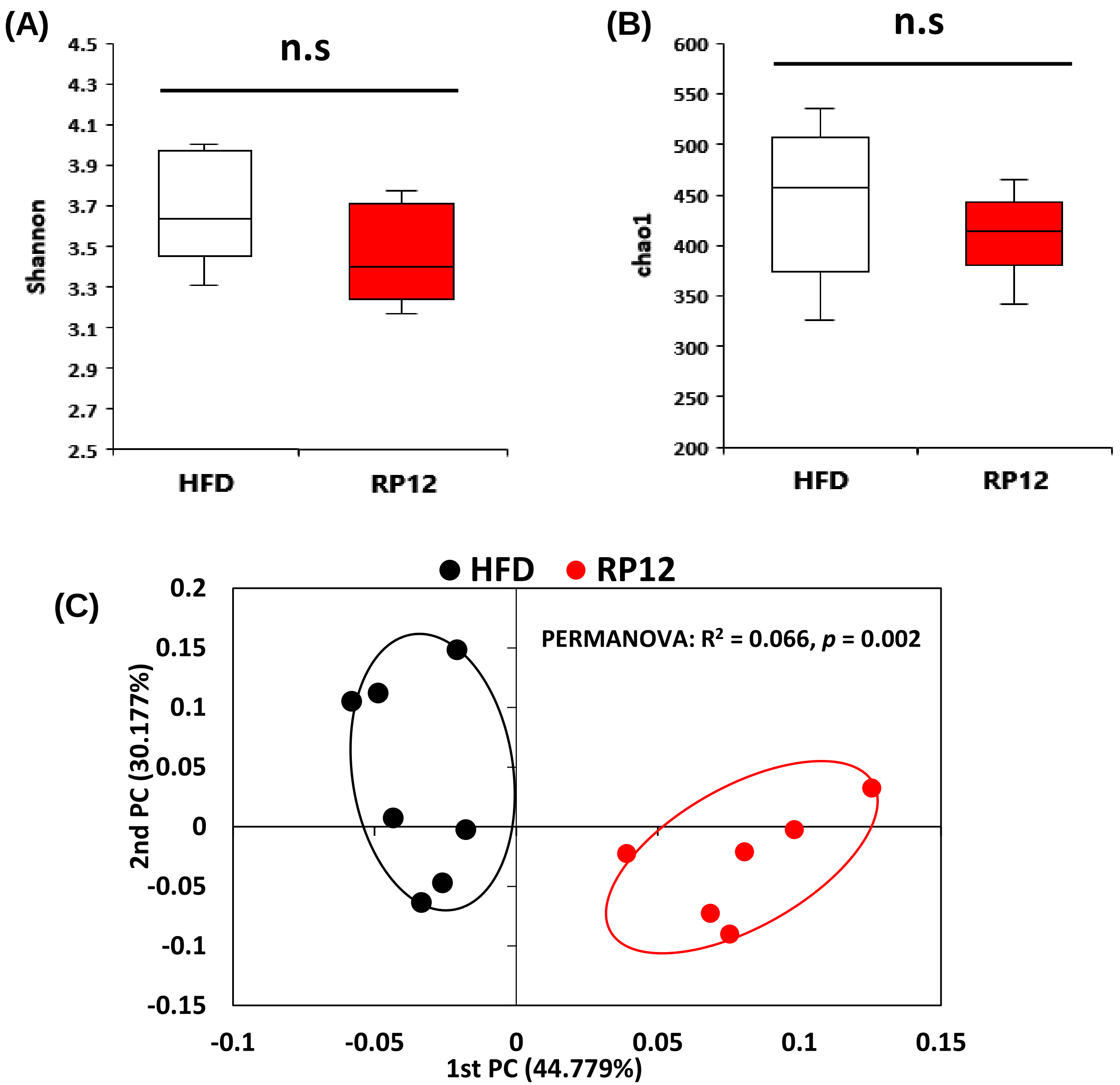

Supplementary Figure 4. Alpha diversity indexes and PCoA between HFD and RP12 groups. (A) Shannon's diversity index. (B) The Chao1 richness estimator. (C) Fecal microbiota composition of groups is shown by generalized UniFrac principal coordinates analysis (PCoA). Results are shown as mean ± SEM (n = 7 per group). Statistical significance of group separation was confirmed by PERMANOVA (R^2 =0.066, *p*=0.002)

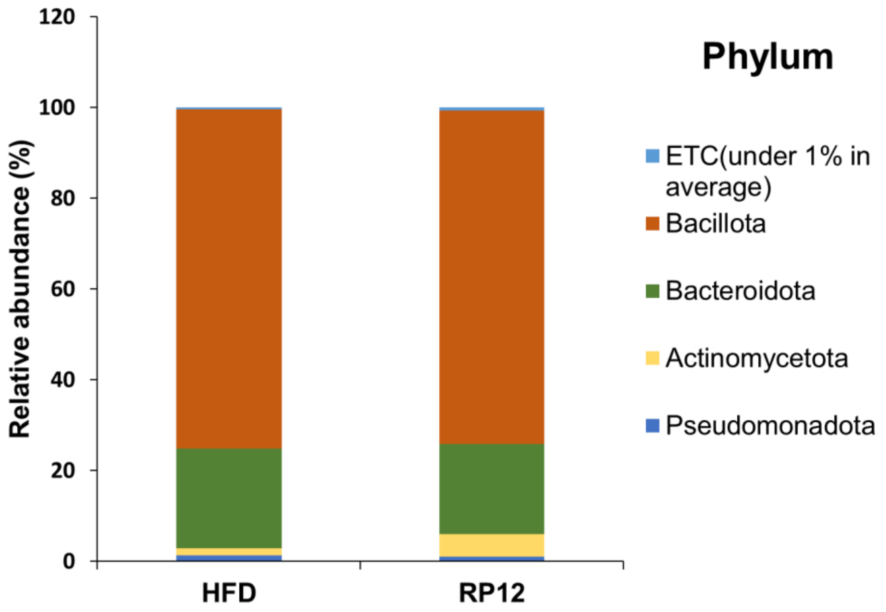

Supplementary Figure 5. Effect of administered *Lactiplantibacillus plantarum* RP12 in the relative abundance of at the phylum on fecal microbiome composition. Results are shown as mean ± SEM (n = 7 per group).

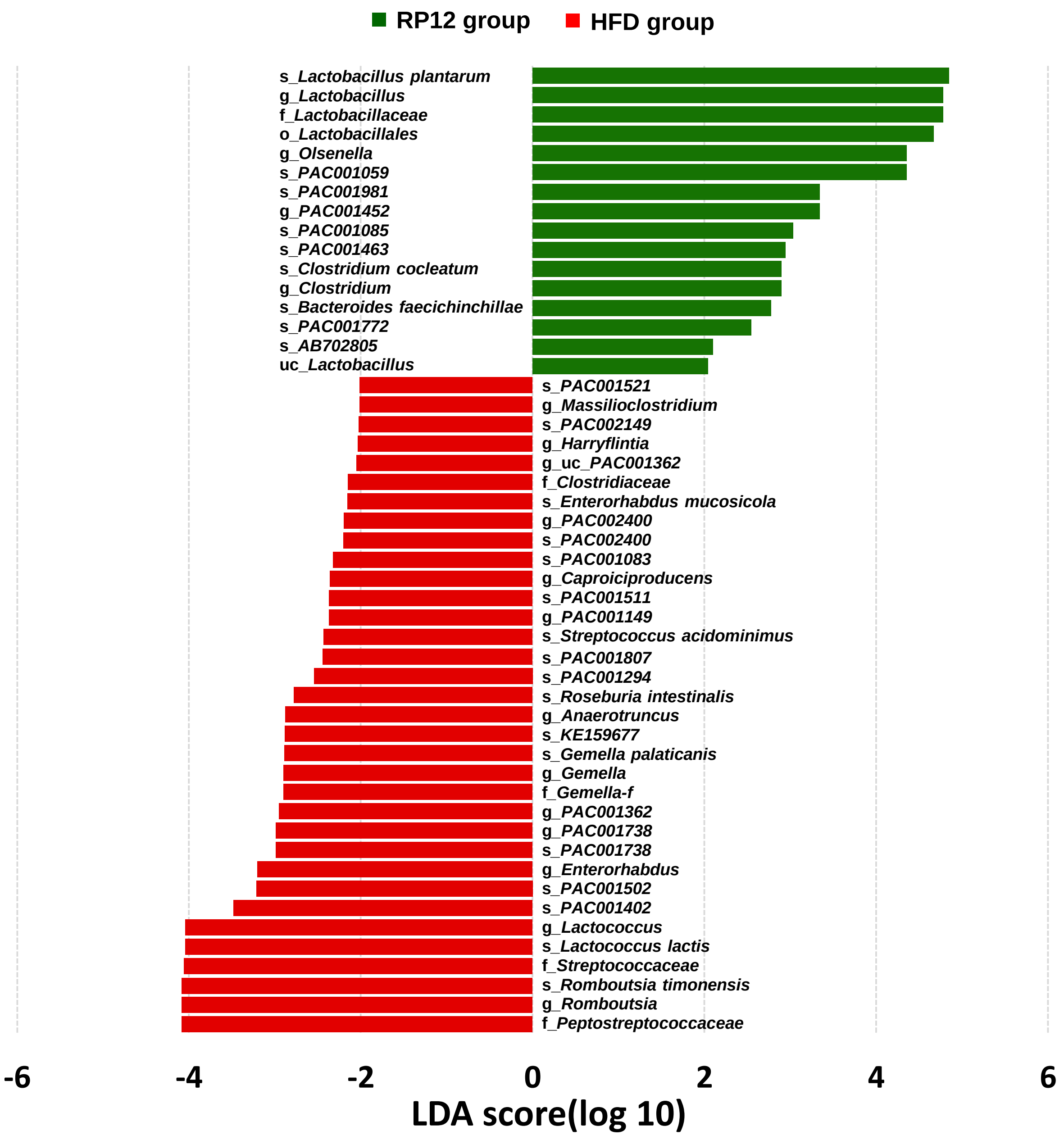

Supplementary Figure 6. Differentially represented genus and species between RP12 and HFD groups through linear discriminant analysis effect size (LEfSe) analysis. Latent Dirichlet allocation (LDA) score indicates the effect size. n = 7 per group

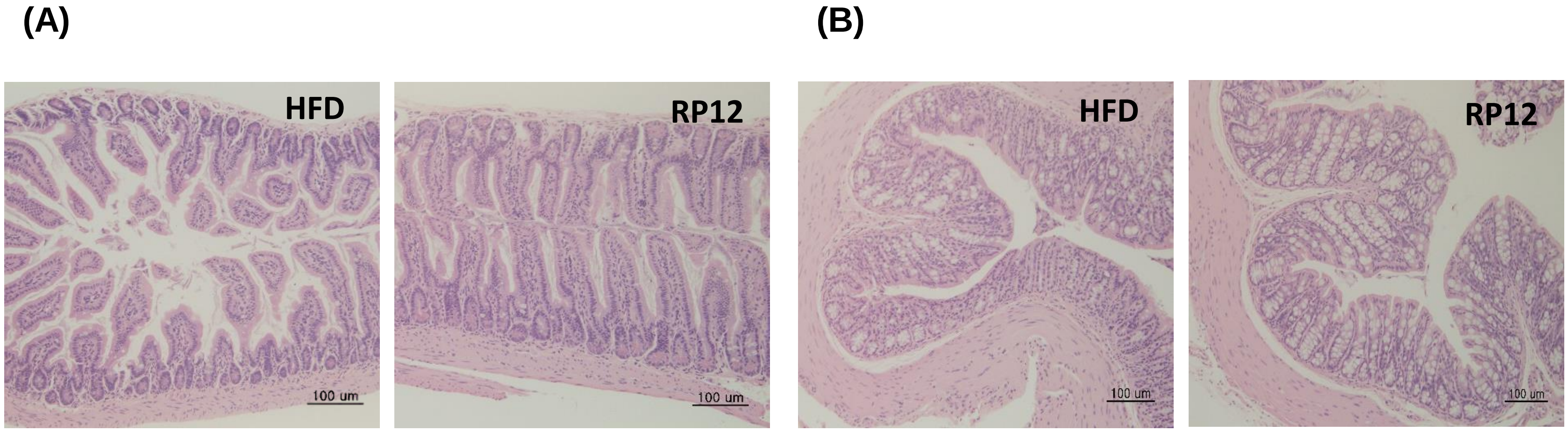

Supplementary Figure 7. Effect of administered *Lactiplantibacillus plantarum* RP12 on the histological parameters in ileum and colon. (A) Representative H&E staining for sections of ileum. Scale bar, =100μm. (B) Representative H&E staining for sections of colon. Scale bar, =100μm.

Supplementary Table 1. Primer sequences for genes used in this study.

Gene	**Forward primer sequence (5′ - 3′)**	**Reverse primer sequence (5′ – 3′)**
Pparγ	CCAGAGCATGGTGCCTTCGC	CAGCAACCATTGGGTCAGCTC
Cebpα	GAACAGCAACGAGTACCGGGTA	GCCATGGCCTTGACCAAGGAG
aP2	CACCGCAGACGACAGGAAG	GCACCTGCACCAGGGC
Cd36	GGCCAAGCTATTGCGACAT	CAGATCCGAACACAGCGTAGA
Lpl	TGGAGAAGCCATCCGTGTG	TCATGCGAGCACTTCACCAG
Srebp-1c	GGTTTTGAACGACATCGAAGA	CGGGAAGTCACTGTCTTGGT
Fas	GCTGCTGTTGGAAGTCAGC	AGTGTTCGTTCCTCGGAGTG
Scd1	GTGCTGGTTGTTGTGCTG	AACTTATCTCCTCCATTCTGC
Tnfα	GCCACCACGCTCTTCTGCCT	GGCTGATGGTGTGGGTGAGG
Mcp1	TCTGGACCCATTCCTTCTTG	AGGTCCCTGTCATGCTTCTG
Il-6	CAAGAAAGACAAAGCCAGAGTCCTT	TGGATGGTCTTGGTCCTTAGCC
Pparα	ATGCCAGTACTGCCGTTTTC	CCGAATCTTTCAGGTCGTGT
Cpt1α	GATGTGGACCTGCATTCCTT	TCTTGTAATGTGCGAGCTG
Ucp2	AACAGTTCTACACCAAGGGC	AGCATGGTAAGGGCACAGTG
Aox1	AGCCATTGACATAGGCCAGA	TCTGGCCTATGTC AATGGCT
Acot1	GTGGCCACCCTGAGGTAAAA	TCTCAGGATAGTCACAGGGGG
Claudin-1	GCGCGATATTTCTTCTTGCAGG	TTCGTACCTGGCATTGACTGG
Muc2	TGCCTGGCCCTGTCTTTG	CAGCTCCAGCATGAGTGC
